# Maspin expression in gastrointestinal stromal tumors

**DOI:** 10.1186/1477-7819-8-22

**Published:** 2010-03-26

**Authors:** Saduman Balaban Adim, Gulaydan Filiz, Ozkan Kanat, Omer Yerci, Halil Ozguc, Berna Aytac

**Affiliations:** 1 Department of Surgical Pathology, Uludag University, Faculty of Medicine, Gorukle, Bursa, 16059, Turkey; 2 Department of Medical Oncology, Uludag University, Faculty of Medicine, Gorukle, Bursa, 16059, Turkey; 3 Department of Surgery, Uludag University, Faculty of Medicine, Gorukle, Bursa, 16059, Turkey

## Abstract

**Background:**

To investigate the role of maspin expression in the progression of gastrointestinal stromal tumors, and its value as a prognostic indicator.

**Methods:**

In the study 54 patients with GIST diagnosis were included in Uludag University of Faculty of Medicine, Department of Pathology between 1997-2007. The expression of maspin in 54 cases of gastrointestinal stromal tumor was detected by immunohistochemistry and compared with the clinicopathologic tumor parameters.

**Results:**

The positive expression rates for maspin in the GISTs were 66,6% (36 of 54 cases). Maspin overexpression was detected in 9 of 29 high risk tumors (31%) and was significantly higher in very low/low (78.6%) and intermediate-risk tumors (63.6%) than high-risk tumors.

**Conclusions:**

Maspin expression might be an important factor in tumor progression and patient prognosis in GIST. In the future, larger series may be studied to examine the prognostic significance of maspin in GISTs and, of course, maspin expression may be studied in different mesenchymal tumors.

## Background

Gastrointestinal stromal tumors (GISTs) are the most common mesenchymal tumors of the gastrointestinal tract. These tumors may occur in any region but are most commonly reported in the stomach and the small intestine [1-7]. GISTs originate from the neoplastic transformation of the intestinal pacemaker cell, the interstitial cell of Cajal. Cajal cells are neuron-derived cells that migrate from the neural crest to the intestine, and the GISTs stemming from these are different from classical mesenchymal tumors such as leiomyoma [8].

GISTs have a wide spectrum of biological behavior ranging from benign to malignant [1,2]. Despite clearly defined conventional histological criteria such as tumor size and mitotic index, the prediction of the clinical course of these tumors is often difficult [6]. Therefore it is important to investigate alternative markers that allow better prognostic assessment.

Maspin (mammary serine protease inhibitor) is a member of the serpin superfamily of protease inhibitors, which also acts as a tumor suppressor [9]. The mechanism of its tumor suppressor effect is still not understood clearly. It is suggested that maspin prevents invasion and metastases of tumors by inhibiting tumor-induced angiogenesis and tumor cell motility [10-13]. In addition, it is reported to induce apoptosis of neoplastic cells [14]. Maspin expression has been demonstrated in multiple tissues including breasts, prostate, placenta, small intestine, colon, uterus, kidney, thymus, and testis [15-18]. On the other hand, it is expressed at different levels in many solid tumors. In breast, colon, stomach, thyroid, bladder, and prostate cancers, in lung and oral cavity squamous cell carcinoma, and in certain renal neoplasms, maspin expression seems to predict a better prognosis [13,19-22]. In contrast, some studies have shown that maspin overexpression is correlated with a poor prognosis in pancreatic and ovarian cancers and in lung adenocarcinoma [23-25].

To the best of our knowledge, no study yet exists about the presence of maspin expression in mesenchymal and neural tumors. On the other hand, existing studies report that maspin expression does not occur in mesenchymal tissue other than corneal stromal cells. Similarly, GISTs have not yet been studied in relation to maspin expression and prognostic meaning. The aim of this study was an immunohistochemical evaluation of maspin expression in these tumors.

## Methods

Between 1997 and 2007, fifty-four patients with GIST who underwent surgical resection in Uludag University of Faculty of Medicine were selected for this study. Seven of the patients had distant metastasis at the time of presentation. Ethical approval was obtained for the study. All specimens of the 54 patients showed positive CD117 and/or CD34 immunostaining. According to the classification system proposed by Fletcher et al. (Table 1), 29 (53.7%) patients belonged to the high-risk group. Forty-two (77.8%) patients had tumors ≥ 5 cm, and 32 (59.2%) had mitotic counts ≥ 5/50 high power fields (HPF).

**Table 1:** Risk of Aggressive Behavior in GISTs (Fletcher et al, 2002)

	Size (largest dimension)	Mitotic Count
Very low risk	<2 cm	< 5/50 HPF

Low risk	2-5 cm	< 5/50 HPF

Intermediate risk	<5 cm	6-10/50 HPF
	
	5-10 cm	< 5/50 HPF

High risk	>5 cm	> 5/50 HPF
	
	>10 cm	any mitotic rate

### Immunohistochemistry

The cellular expression of maspin was assessed by immunohistochemistry (IHC) using specific antibody on routinely processed blocks of formalin-fixed and paraffin-embedded surgical specimens of the tumors. The 4 μm sections of tumor tissues were mounted on poly-L-lysine coated slides. The sections were deparaffinized in xylene (25 min) and rehydrated through serial baths of alcohol to water. Antigen retrieval was applied with pressure cooking using 500 ml 1 mM diluated EDTA-Saline buffer (pH = 8). After using H_2_O_2 _treatment for 15 minutes to remove endogenous peroxidase activity, nonspecific blockage with ultrablock nonspecific blocking agent (Labvision Co.) was performed on all sections for 10 minutes. Then the sections were incubated with primary antibody maspin AB-1 (Clone EAW24, Mouse monoclonal antibody, Thermo Scientific, USA) 1/20 in dilution at room temperature for 30 minutes. The antibody-treated slides were rinsed in phosphate-buffered saline solution and incubated with a biotinylated secondary antibody (Ultravision-Labcision Co., Fremont, CA, U.S.A.). The slides were washed in phosphate-buffered saline and then incubated with an avidin-biotin-preoxidase complex (Ultra-streptavidin/HRP, Labvision Co.) for 30 minutes. As a chromogen, 3-3'-diamino-benzene tetrahydrochloride was used with hydrogen peroxide. The slides were finally counterstained with hematoxylin. Prostate tissue was used as positive control.

### Evaluation of staining for Maspin

Maspin expression was determined semiquantitatively by the percentage of stained cells, the staining intensity, and subcellular localization [23].

• The percentage of positive cells was rated as follows: 0 points, no positive cells; 1 point, 0-5%; 2 points, 6-50%; 3 points, 50-100% positive cells.

• Staining intensity was rated as follows: 1 point, weak intensity; 2 points, moderate intensity; 3 points, strong intensity.

• Points for intensity and the percentage of positive cells were added to obtain an overall maspin score (OMS) [0-3]. Lesions were categorized into four groups:

1) Negative (OMS = 0): < 5% stained cells regardless of intensity

2) Weak expression (OMS = 1): 3 points

3) Moderate expression (OMS = 2): 4-5 points

4) Strong expression (OMS = 3): 6 points

• OMS 2 & 3 was considered as overexpression.

### Statistical analysis

All statistical analyses were performed using SPSS (Statistical Package for the Social Sciences, Chicago, IL) for Windows version 15.0. Survival time was calculated starting from the date of initial surgery.

The Chi-square and Fisher's exact tests were used to evaluate correlations between variables. *P *< 0.05 was considered statistically significant. Survival times were calculated by using the Kaplan-Meier method and compared with the log-rank test.

## Results

Of the 54 patients included in this study, 35 (64.8%) were male and 19 (35.2%) were female (Table 2). The mean age was 55.8 years (range 17-75 years). Tumor localizations were as follows: 23 stomach (42.6%), 18 small intestine (33.3%), 10 mesentery (18.5%), 2 large intestine (3.7%), and 1 esophagus (1.85%). The sizes of tumor varied between 2 and 29 cm (median 7 cm). Mitotic count varied between 0 and 61 mitoses per 50 HPF (median 11).

**Table 2:** Clinicopathological characteristics of GIST patients

Variable	n
Age, yr	55,83 ± 12,93

Gender (Male/Female)	35/19

Tumor size	

5 cm <	12

5 cm ≥	42

Mitosis (50 HPP)	

5 <	22

5 ≥	32

Localization	

Stomach	23

Small intestine	18

Large intestine	2

Meso-peritoneum	10

Eusophagus	1

Risk Group	

Low (very low and low)	14

Intermediate	11

High	29

**Figure 1:**
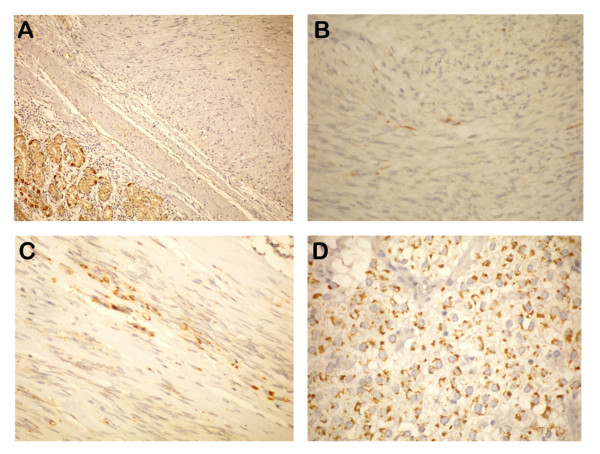
**A) OMS score (-), B) OMS score (1+), C) OMS score (2+), D) OMS score (3+) staining paterns in GIST's**.

Among 54 tumors, 18 were OMS = 0; 9 were OMS = 1; 8 were OMS = 2; and 19 were OMS = 3 (Figure 1). When risk groups were compared, a meaningful difference was observed between low- and high-risk groups, as well as between intermediate- and high-risk groups (chi square test: P = 0,009; P = 0,029)(Table 3).

**Table 3:** Correlation of clinicopathological variables with maspin overexpression.

		Maspin overexpression		
**Variable**	**54 (%)**	**No (n = 27)** **(% 50)**	**Yes (n = 27)** **(% 50)**	**P value**

Size				0,769

5 cm <	12	6	6	

5 cm ≥	42	21	21	

Mitotic count (50 BB)				0,525

5 <	22	7	15	

5 ≥	32	20	12	

Risk Group (compared with high risk group and others)				

Very low and low	14	3	11	0,009

Intermediate	11	4	7	0,029

High	29	20	9	

Follow-up period ranged between 2 and 115 months (median: 40.77 months). At the end of the follow up, 37 patients were alive without any evidence of the disease, while 16 had died of the disease and one patient with benign GIST had died of acute pancreatitis. At the time of diagnosis, metastasis was seen in 7 of the high risk tumor patients, and not in others. Overexpression was observed in only 1 of these 7 patients. The maspin +/- ratio in the 47 non metastatic patients was 21/26 (p = 0,050).

Overall mean survival length was 54.67 months. It was 56.19 months in maspin (+) patients and 53.29 months in maspin (-) patients. There was no difference in survival rates between the groups (p > 0.05).

## Discussion

GISTs are a rare special mesenchymal tumor group, making up less than 1% of primary tumors of the gastrointestinal system [1]. Their biological behavior is hard to predict [2]. Many macroscopic and microscopic parametres have been suggested to identify the prognosis, including tumor localization and diameter, invasion of peripheral tissue, growth pattern, mucosal invasion, predominant tumor cell type, cellularity, nuclear pleomorphism, mitotic count, Ki67 proliferative activity index, p53 gene mutation, histological grade, DNA analysis, margins of surgical operation, necrosis and immunophenotyping [1-7]. Even though efforts continue for the identification of new parameters, tumor diameter and mitotic index (mitotic count/50 BBA) currently remain the most important morphological criteria for the prediction of tumor behavior [2,6].

Proteinases and proteinase inhibitors are known to play an important role in tumor invasion and metastasis. Proteinases degrade the extracellular matrix, while their inhibitors antagonize this process. Two classes of proteinases have been extensively studied: serine proteinases and their inhibitors, and metalloproteinases and their inhibitors [26]. Maspin (mammary serine protease inhibitor) is structurally a member of the serpine (serine protease inhibitors) superfamily [27]. Studies have revealed that maspin is largely an intracellular protein, which is soluble in the cytoplasm and associated with secretory vesicles. It is located at the cell membrane interface with extracellular matrix and does not act as a classical inhibitory serpine with antiprotease activity against trypsin-like proteases [28].

Maspin expression has been shown in the literature in epithelial and myoepithelial cells in certain tissues, most notably in the breasts and prostate, as well as neoplasms stemming from these tissues [15-18]. Many articles have described a negative association between maspin expression and carcinoma progression in several malignancies, including those of the breast, prostate, colon, bladder, thyroid and stomach cancers, lung and oral cavity squamous cell carcinoma, and some renal neoplasms [13,19-22]. Chen and Yates reported that maspin has suppressive effects on invasion and metastasis of carcinoma [29]. However, enhanced maspin expression may have an impact on different steps in the progression to pancreatic and ovarian carcinoma [23-25]. In addition, different results were also obtained for the same cancer type in different studies [21,30]. These contradictory results might result from specific regulation in different organs or the different genetic backgrounds of the populations studied, although definite evidence for a paradoxical mechanism remains elusive.

While existing studies report that maspin expression does not occur in mesenchymal and neural crest cells other than corneal stromal cells, the literature does not include any studies about maspin expression in mesenchymal and neural tumors, except gliomas [18,31,32]. Similarly, no study exists to show the relationship between maspin expression and prognosis in GIST patients.

Although the molecular and biological mechanisms of the function(s) of maspin remain largely unknown at present, there is evidence that maspin interacts with the p53 tumor suppressor pathway and may function as inhibitor to cell motility, invasion, metastasis and angiogenesis in vitro and in vivo [23]. Also, maspin appears to be regulated by wild-type p53. Zou et al. reported that there was robust induction of maspin in prostate and breast cancer cells after wild-type p53 expression [33]. p53 was found to activate maspin promoter by binding directly to the p53 consensus binding site present in the maspin promoter. Some GISTs are known to harbor p53 gene mutations [34,35].

**Figure 2:**
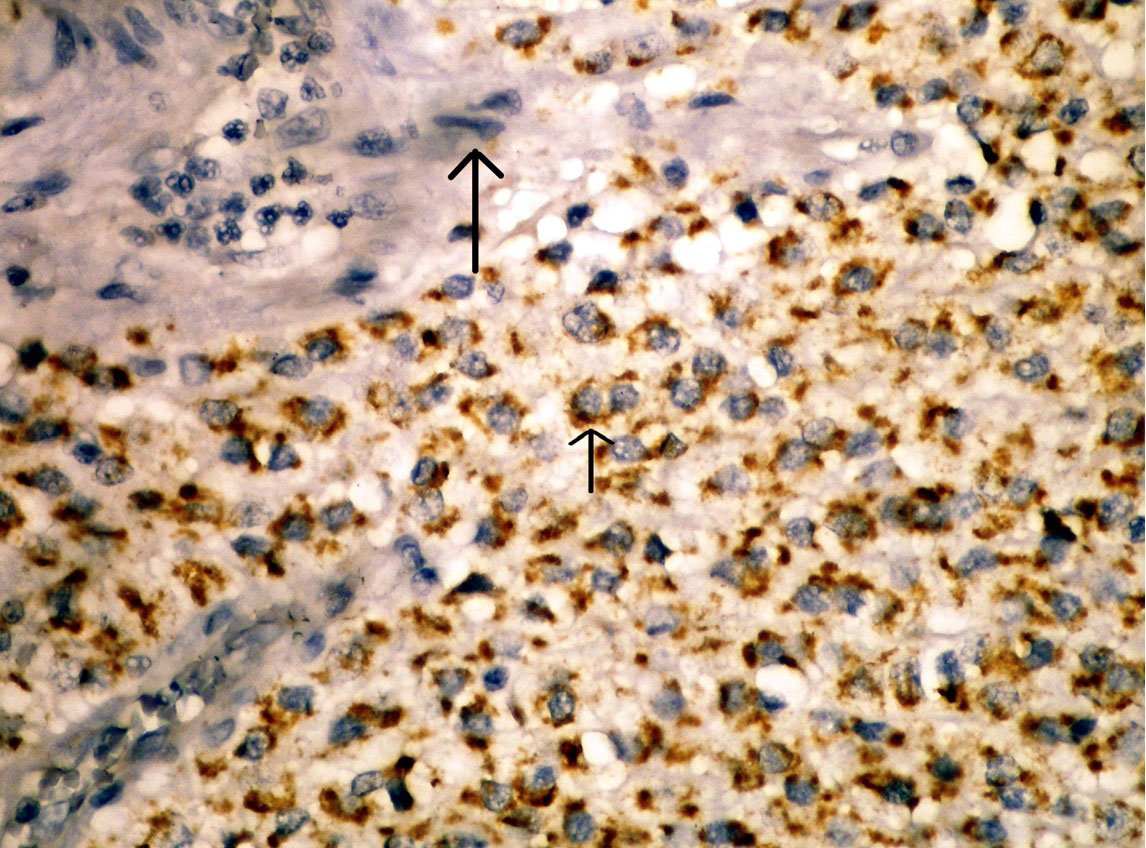
**Positive staining in tumor cell's cytoplasm for maspin (short arrow) but not stain in endothelial cells (long arrow)**. (×200)

In this study, we primarily studied the existence of maspin expression in GIST cases. We immunohistochemically observed maspin staining in 36 (66,6%) out of our 54 cases. In half of these patients, we obtained 5% or more positive cytoplasmic staining with maspin, whereas in 16,6% of them we obtained less than 5% staining. In the blocks we studied, we detected cytoplasmic staining with maspin in the mucosa epithelium of normal tissues belonging to the gastrointestinal tract neighboring the tumor, but no staining in mesenchymal cells (smooth muscle cells, endothelial cells, etc.). No staining occurred in the endothelial cells of tumor tissue either (Figure 2). Following these, we analyzed the prognostic significance of maspin expression in GISTs.

In our study, maspin expression was significantly correlated with the risk grade of GISTs. Maspin overexpression was detected in 9 of 29 high risk tumors (31%) and was significantly higher in very low/low- (78.6%) and intermediate-risk tumors (63.6%) than high-risk tumors.

As low-risk patients have higher maspin overexpression and high-risk patients have less, it may be claimed that maspin overexpression is a favorable prognosis marker.

## Conclusions

Our preliminary results suggest that expression level of maspin may be considered a predictor of prognosis in GISTs. Future studies with larger patient numbers will be essential to confirm the prognostic significance of maspin in patients with GIST and other mesenchymal tumors.

## Competing interests

The authors declare that they have no competing interests.

## Authors' contributions

SBA designed the study, researched the literature, and drafted the manuscript. SBA, GF, OY, and BA contributed to the histopathological analyses. OK and HO participiated in the study design and coordination, and helped to collect data.
